# USP45-mediated deubiquitination of HIV-1 Tat regulates viral transcription and latency

**DOI:** 10.3389/fmicb.2026.1656512

**Published:** 2026-01-26

**Authors:** Kei Miyakawa, Hiyori Okura, Yasuyoshi Hatayama, Mayuko Nishi, Hirokazu Kimura, Hiroyuki Yamamoto, Hideki Hasegawa, Tetsuro Matano, Akihide Ryo

**Affiliations:** 1 AIDS Research Center, National Institute of Infectious Diseases, Japan Institute for Health Security, Tokyo, Japan; 2 Influenza Research Center, National Institute of Infectious Diseases, Japan Institute for Health Security, Tokyo, Japan; 3 Department of Microbiology, Yokohama City University School of Medicine, Kanagawa, Japan; 4 Institute for Vaccine Research and Development, Hokkaido University, Hokkaido, Japan; 5 Department of Bioinformatics and Integrative Omics, National Institute of Infectious Diseases, Japan Institute for Health Security, Tokyo, Japan; 6 School of Medical Technology, Faculty of Health Sciences, Gunma Paz University, Gunma, Japan; 7 Institute of Medical Science, University of Tokyo, Tokyo, Japan

**Keywords:** deubiquitination, HIV-1, interferon-stimulated gene, tat protein, viral latency

## Abstract

The HIV-1 Tat protein is regulated by post-translational modifications, particularly ubiquitination, but the full spectrum of deubiquitinating enzymes (DUBs) controlling HIV-1 transcription remains incompletely understood. We developed a NanoBRET-based screening system using a library of 120 DUB expression vectors and identified USP45 as a novel Tat-interacting DUB. Functional characterization revealed that USP45 specifically deubiquitinates Tat at lysine 19, targeting both K48-linked and K63-linked ubiquitin chains. Notably, USP45 overexpression suppressed Tat-dependent transcriptional activation and HIV-1 viral particle production, while USP45 knockdown enhanced both processes. Transcriptional profiling in latently infected J-Lat 8.4 cells using digital PCR revealed that USP45 primarily inhibits the initial stages of viral transcription, thereby contributing to the maintenance of the latent state by restricting downstream transcriptional processes. Furthermore, USP45 expression is induced by interferons, identifying it as an interferon-stimulated gene. These findings establish that USP45 functions as a host restriction factor that negatively regulates HIV-1 transcription by promoting Tat deubiquitination at lysine 19, representing a promising therapeutic target for controlling HIV-1 latency.

## Introduction

The function of proteins is precisely regulated through various post-translational modifications. Ubiquitination, in particular, is a modification in which the 76-amino acid protein ubiquitin is covalently attached to lysine residues of target proteins (Komander and Rape, 2012). K48-linked polyubiquitin chains generally mediate protein degradation, while K63-linked chains are responsible for degradation-independent functional regulation. Ubiquitination is a reversible process. Deubiquitinating enzymes (DUBs) remove ubiquitin from target proteins. Humans possess approximately 100 DUBs that precisely control protein stability, localization, and function (Nijman et al., 2005; Reyes-Turcu et al., 2009). DUBs are implicated in various pathological conditions, including cancer, neurodegenerative diseases, and viral infections, making them attractive therapeutic targets (He et al., 2016).

The HIV-1 Tat protein is a multifunctional transcription factor essential for viral transcriptional activation. Tat forms a complex with Cyclin T1 and CDK9 and binds to HIV-1 TAR RNA to promote viral genome transcription (Wei et al., 1998; Garber et al., 2000). Interestingly, HIV-1 Tat ubiquitination exhibits complex dual functionality that differs from typical degradation signals. While K48-linked ubiquitination subjects Tat to proteasome-dependent degradation, K63-linked ubiquitination and ubiquitination at lysine 71 by the Hdm2 E3 ligase have been reported to serve non-degradative functions that enhance Tat transcriptional activity (Bres et al., 2003; Hetzer et al., 2005; Faust et al., 2017). Recent studies have revealed that multiple DUBs regulate Tat function during HIV-1 infection, with some promoting viral replication while others act as restriction factors (Ali et al., 2017; Xu et al., 2018; Gao et al., 2021). However, the full spectrum of DUBs controlling HIV-1 transcription remains incompletely understood.

HIV-1 latency represents the greatest barrier to anti-HIV therapy. In latently infected cells, although viral DNA is integrated into the host genome, transcription is suppressed, and viral production does not occur (Hakre et al., 2011). Reduced Tat transcriptional activity is intimately associated with this latent state, and elucidating its regulatory mechanisms is crucial for developing curative therapeutic strategies (Hakre et al., 2011; Rice, 2017). However, DUB regulation of Tat in HIV-1 latency remains insufficiently understood. Furthermore, the DUBs identified to date are limited, and it is highly likely that additional DUBs involved in the complex ubiquitination regulation of Tat exist.

In this study, we aimed to elucidate novel molecular mechanisms involved in HIV-1 transcriptional regulation and latency through the development and application of a novel screening system for comprehensive identification of DUBs that interact with HIV-1 Tat.

## Results

### Identification of DUBs that interact with HIV-1 Tat protein

**Figure 1:**
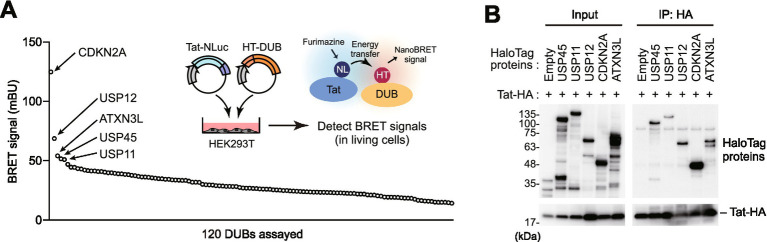
Comprehensive screening identifies DUBs that interact with HIV-1 Tat protein. **(A)** NanoBRET screening results of 120 DUB expression vectors for interaction with HIV-1 Tat. HEK293T cells were co-transfected with NanoLuc-fused Tat and HaloTag-fused DUBs. The top five candidates (CDKN2A, USP12, ATXN3L, USP45, and USP11) are highlighted. Data represent mean from two independent experiments. **(B)** Validation of Tat-DUB interactions by immunoprecipitation. HEK293T cells were co-transfected with HA-tagged Tat and the indicated DUB constructs. Cell lysates were immunoprecipitated with anti-HA antibody, and co-precipitated DUBs were detected by Western blotting.

To identify deubiquitinating enzymes (DUBs) that interact with HIV-1 Tat, we constructed a library of 120 HaloTag-fused DUB-related gene expression vectors and performed comprehensive screening using the NanoBRET method. The NanoBRET assay is a technique that utilizes bioluminescence resonance energy transfer (BRET) between a donor (NanoLuc luciferase) and acceptor (HaloTag) to quantitatively detect protein–protein interactions in real time. In this experiment, we used HEK293T cells co-expressing NanoLuc-fused Tat protein and HaloTag-fused DUBs, evaluating interactions based on increased BRET signal intensity. Screening of the 120 DUB library identified the top five candidates showing high BRET signal: CDKN2A, USP12, ATXN3L, USP45, and USP11 (Figure 1A). We validated the interactions of these candidates using immunoprecipitation experiments with HA-tagged proteins, confirming that all candidates specifically co-precipitated with Tat-HA (Figure 1B). Among these, interactions between CDKN2A and USP11 with Tat have been previously reported (Gargano et al., 2008; Jager et al., 2011; Boehm et al., 2023), demonstrating the validity of our screening system.

### USP45 mediates Tat deubiquitination

**Figure 2:**
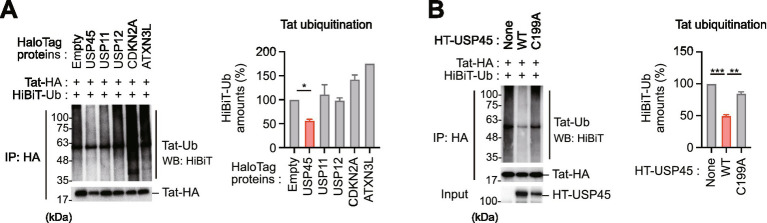
USP45 mediates enzymatic activity-dependent deubiquitination of Tat protein. **(A)** Deubiquitinating activity of candidate DUBs toward Tat protein. HEK293T cells were co-transfected with HA-tagged Tat, HiBiT-tagged ubiquitin, and the indicated HaloTag-fused DUBs. Tat ubiquitination levels were assessed by immunoprecipitation with anti-HA antibody followed by Western blotting with HiBiT detection. Quantification shows relative HiBiT-ubiquitin levels normalized to empty vector control. **(B)** Catalytic activity requirement for USP45-mediated Tat deubiquitination. HEK293T cells were transfected with HA-tagged Tat, HiBiT-tagged ubiquitin, and either wild-type USP45 or catalytically inactive USP45 C199A mutant. Data represent mean ± SD from three independent experiments. *p < 0.05, **p < 0.01, ***p < 0.001.

To identify the DUBs involved in Tat deubiquitination, we next performed an immunoprecipitation analysis using cell extracts co-expressing HA-tagged Tat and HiBiT tag-fused ubiquitin, which enables the quantitative measurement of ubiquitination levels (Ranawakage et al., 2019). Our results revealed that HEK293T cells overexpressing USP45, particularly among the candidate DUBs, showed approximately 50% reduction in Tat ubiquitination (Figure 2A). In contrast, the USP45 C199A mutant, in which a mutation was introduced into the catalytic active site (Conte et al., 2018), completely lost Tat deubiquitinating activity (Figure 2B). To further determine which type of polyubiquitin chains USP45 targets, we performed immunoblot analysis using K48-specific and K63-specific antibodies. Interestingly, USP45 was found to promote deubiquitination of both K48-type and K63-type ubiquitin chains (Supplementary Figure S1).

### USP45 suppresses Tat transcriptional activity

**Figure 3:**
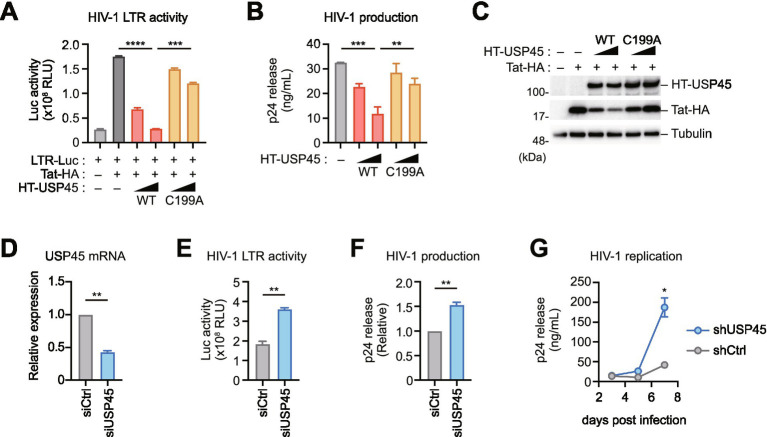
USP45 suppresses Tat transcriptional activity and HIV-1 replication. **(A)** Effect of USP45 overexpression on HIV-1 LTR-driven transcription. HEK293T cells were co-transfected with HIV-1 LTR-luciferase reporter, Tat-HA, and increasing amounts of wild-type or catalytically inactive (C199A) USP45. Luciferase activity was measured 48 h post-transfection. ***p < 0.001. **(B)** USP45 reduces HIV-1 particle production. HEK293T cells were transfected with HIV-1 proviral DNA and increasing amounts of USP45 (wild-type or C199A). Viral production was assessed by p24 ELISA of culture supernatants at 48 h post-transfection. **p < 0.01, ***p < 0.001. **(C)** USP45 overexpression decreases Tat protein levels. Western blot analysis of cells from (A) expressing USP45 (WT or C199A) and Tat-HA. **(D-G)** Effects of endogenous USP45 knockdown on HIV-1 replication. RT-qPCR analysis confirming approximately 60% USP45 knockdown efficiency using specific siRNA **(D)**. USP45 knockdown enhances HIV-1 LTR transcriptional activity **(E)** and viral particle production **(F)** compared to control siRNA. Long-term replication assay in Jurkat T cells with stable USP45 knockdown (shUSP45) shows enhanced HIV-1 replication **(G)**. Statistical comparisons were made against the control cells. *p < 0.05, **p < 0.01.

To delineate the impact of USP45 on Tat function, we measured Tat-dependent transcriptional activity using a luciferase reporter vector containing the HIV-1 LTR promoter. Our results showed that USP45 overexpression significantly reduced Tat-dependent LTR activity, whereas the catalytically inactive C199A mutant exhibited significantly weaker suppression, demonstrating the requirement for deubiquitinase activity (Figure 3A). Additionally, USP45 reduced HIV-1 production efficiency in an enzymatic activity-dependent manner (Figure 3B). Interestingly, USP45 overexpression decreased Tat expression levels, suggesting a potential impact on intracellular stability (Figure 3C). Cycloheximide chase experiments confirmed that wild-type USP45 accelerated Tat protein degradation, while C199A failed to promote degradation, allowing stabilization of ubiquitinated Tat (Supplementary Figure S2). These results indicate that USP45-mediated deubiquitination reduces intracellular stability and suppresses Tat function.

To examine the effects of endogenous USP45, we transduced HEK293T cells with USP45 knockdown using specific siRNA. Although multiple siRNAs were used, only approximately 50% knockdown efficiency was achieved (Figure 3D; Supplementary Figure S3A). When Tat-dependent transcriptional activity was examined in these USP45 knockdown cells, HIV-1 transcriptional activity increased 2-fold compared to controls (Figure 3E). Concomitantly, HIV-1 particle production also increased (Figure 3F). Furthermore, stable USP45 knockdown in Jurkat T cells via lentiviral shRNA significantly enhanced HIV-1 replication by day 7 post-infection (Figure 3G; Supplementary Figure S3B). These results demonstrate that endogenous USP45 negatively regulates Tat function, thereby suppressing HIV-1 production and replication.

### USP45 mediates deubiquitination of Tat at the lysine 19 residue

**Figure 4:**
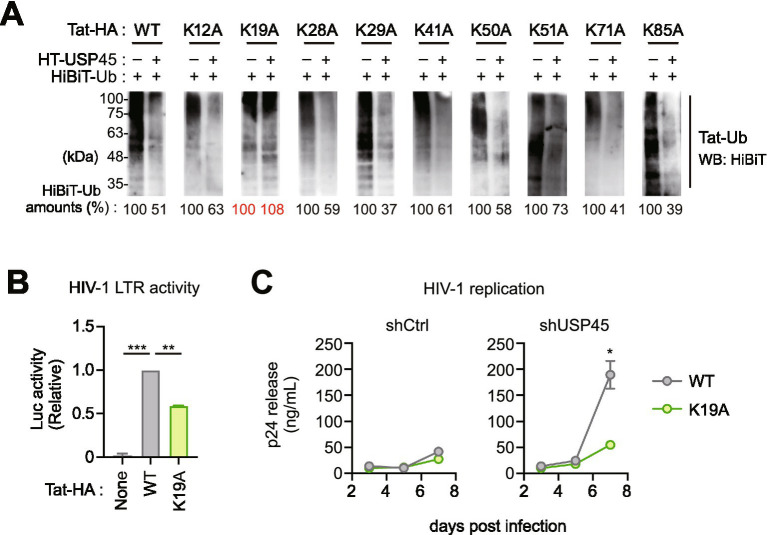
USP45 specifically targets Tat lysine 19 residue for deubiquitination. **(A)** Mapping of USP45 target site on Tat protein. HEK293T cells were co-transfected with wild-type or lysine-to-alanine mutant Tat-HA constructs, HiBiT-tagged ubiquitin, and USP45. Tat ubiquitination was assessed by immunoprecipitation followed by HiBiT detection. Quantification shows relative ubiquitin levels normalized to the respective controls without USP45. **(B)** Functional importance of Tat K19 in transcriptional activity. HIV-1 LTR-luciferase reporter assay of cells expressing Tat (WT or K19A). **p < 0.01, ***p < 0.001. **(C)** The K19A mutation reduces USP45-dependent enhancement of viral replication. Jurkat T cells with stable USP45 knockdown were infected with wild-type or K19A mutant HIV-1. p24 levels were measured by ELISA over 7 days post-infection. *p < 0.05.

The HIV-1 Tat protein contains nine lysine residues (K12, K19, K28, K29, K41, K50, K51, K71, K85) that can serve as ubiquitination targets. To identify the target sites for USP45-mediated deubiquitination, we generated Tat mutants with each lysine residue substituted with alanine and compared the efficiency of USP45-mediated deubiquitination. Our results showed that only the K19A mutant exhibited resistance to USP45-mediated deubiquitination (Figure 4A). To confirm that K19 is indeed ubiquitinated, we performed ubiquitination assays and found that the K19A mutant showed substantially reduced ubiquitination compared to wild-type Tat (Supplementary Figure S4A) demonstrating that K19 is one of the major ubiquitination sites. These results indicate that the K19 residue is the primary target for USP45-mediated deubiquitination.

To validate the biological significance of USP45-mediated Tat K19 deubiquitination, we generated a mutant virus by introducing the Tat K19A mutation into HIV-1 proviral DNA (pNL4-3). Measurement of HIV-1 transcriptional activity using the LuSIV reporter system revealed that the Tat K19A mutant virus showed approximately 50% reduced transcriptional activity compared to wild-type (Figure 4B). Furthermore, when we examined viral replication efficiency in Jurkat T cells at low MOI, while wild-type virus showed a 4.5-fold increase in replication efficiency upon USP45 knockdown, the K19A mutant virus showed only a 2.0-fold increase (Figure 4C). This attenuated response to USP45 depletion was reproducible at higher MOI, confirming that USP45’s suppressive effects are robust across different infection conditions (Supplementary Figure S4B). Additionally, we observed that endogenous USP45 protein levels increased upon HIV-1 infection, indicating its induction as an antiviral factor (Supplementary Figure S3B,C). These results suggest that the suppressive effects of USP45 are primarily mediated through deubiquitination of Tat K19.

### USP45 inhibits multiple stages of HIV-1 transcription in latently infected cells

**Figure 5:**
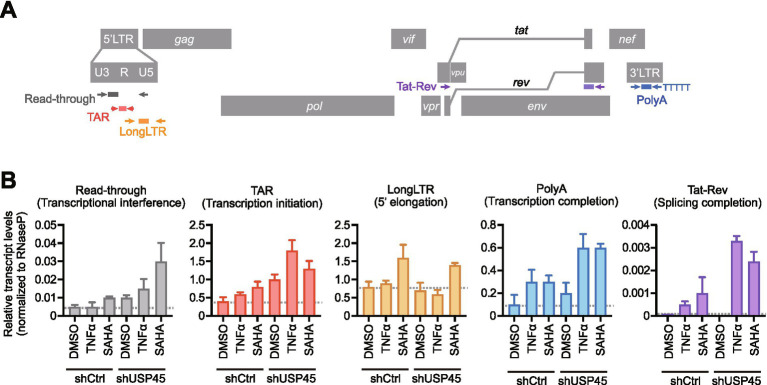
USP45 inhibits early stages of HIV-1 transcription and contributes to latency maintenance. **(A)** Schematic representation of HIV-1 transcriptional profiling assay. The HIV-1 proviral genome is shown with primer/probe locations for detecting five distinct transcriptional products: Read-through transcripts (gray) indicating transcriptional interference, TAR transcripts (red) representing transcriptional initiation, LongLTR transcripts (orange) indicating 5′ elongation, PolyA transcripts (blue) representing transcription completion, and Tat-Rev transcripts (purple) indicating splicing completion. **(B)** Transcriptional profiling of HIV-1 latency reversal in USP45 knockdown cells. J-Lat 8.4 cells with control or USP45 shRNA were treated with latency reversing agents (TNFα 10 ng/ml or SAHA 1 μM) or DMSO control. RNA was extracted and analyzed by RT-dPCR using stage-specific primer sets. Viral transcript levels were quantified by digital PCR and normalized to RNaseP gene expression as an internal control. Data represent mean ± SD from three independent experiments.

To elucidate the role of USP45 in HIV-1 latency and reactivation, we performed analyses using the HIV-1 latently infected model cell line J-Lat 8.4. To quantitatively evaluate the effects of USP45 expression suppression on each stage of HIV-1 transcription, we applied the HIV transcriptional profiling assay by digital RT-PCR (RT-dPCR), which has been established in previous studies (Kaiser et al., 2017; Yukl et al., 2018; Telwatte et al., 2019). We used specific primer-probe sets to quantify five different HIV transcripts by RT-dPCR: read-through transcripts indicating transcriptional interference beyond the 3′ LTR, transcripts that indicate HIV transcriptional initiation (TAR), 5′ elongation (LongLTR), polyadenylated (PolyA), and multiple-splicing (Tat-Rev) (Figure 5A).

Analysis of HIV-1 transcriptional dynamics in USP45 knockdown cells under Latency Reversing Agent treatment (LRAs, TNFα and SAHA) revealed comprehensive effects across multiple stages of viral transcription (Figure 5B). Notably, we observed that TNFα and SAHA treatment increased endogenous USP45 protein levels (Supplementary Figures S5A,B), suggesting a potential cellular feedback mechanism to limit viral reactivation. Despite this LRA-induced USP45 upregulation, TAR transcripts, representing transcriptional initiation, were most markedly elevated in USP45 knockdown cells upon LRA treatment, indicating that USP45 primarily restricts the initial stages of HIV-1 transcription. Following this enhanced initiation, downstream transcriptional processes were also substantially increased: PolyA transcripts and multiply-spliced transcripts were elevated upon USP45 depletion, especially in LRA-treated cells. Additionally, read-through transcripts were increased in the absence of USP45, suggesting impaired transcriptional termination control. While LongLTR showed no significant changes, the coordinated increase across transcriptional stages indicates that USP45 functions as a broad transcriptional repressor of HIV-1 gene expression, with particularly pronounced effects on transcriptional initiation. These findings suggest that USP45 maintains HIV-1 latency through multi-level transcriptional suppression in latently infected T cells.

### USP45 is induced by interferons

**Figure 6:**
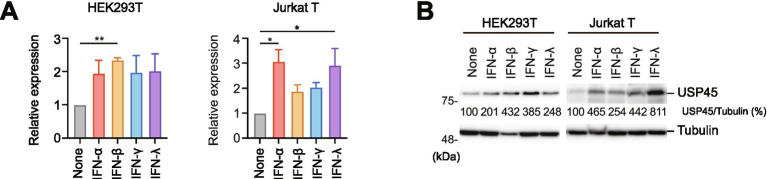
USP45 is induced by interferon treatment. **(A)** USP45 mRNA and **(B)** protein expression levels in HEK293T and Jurkat T cells treated with type I (IFN-α, IFN-β), type II (IFN-γ), or type III (IFN-λ1) interferons for 24 h. mRNA levels were analyzed by qPCR and normalized to untreated controls. Protein expression was analyzed by immunoblotting with tubulin as loading control. Data represent mean ± SD from three independent experiments. *p < 0.05, **p < 0.01.

To elucidate the regulatory mechanisms of USP45 expression, we examined the effects of various interferons on USP45 expression. HEK293T cells and Jurkat T cells were treated with type I (IFN-α/β), type II (IFN-γ), and type III (IFN-λ1) interferons, and USP45 mRNA expression levels were analyzed by qPCR. Results revealed that all tested interferon types enhanced USP45 expression by approximately 2-fold (Figure 6A). Western blot analysis confirmed robust upregulation of USP45 protein in both cell types (Figure 6B). These findings suggest that USP45 acts as an interferon-stimulated gene (ISG), suppressing HIV-1 transcription as part of the host innate immune response.

## Discussion

In this study, we identified that the deubiquitinating enzyme USP45 specifically deubiquitinates the K19 residue of HIV-1 Tat protein and functions as a novel antiviral factor that negatively regulates viral genome transcription and viral replication. The discovery that USP45 is induced by interferons suggests that it functions as an intrinsic antiviral factor by promoting the deubiquitination of the Tat protein, thereby playing a role in the innate immune response. This also indicates that post-translational regulation plays a role in HIV-1 latency, potentially leading to new therapeutic approaches targeting these mechanisms.

Recent studies have revealed that Tat protein is precisely regulated by multiple E3 ligases. UHRF1 promotes K48-linked ubiquitination of Tat and maintains HIV-1 latency by competitively inhibiting its binding to P-TEFb (Liang et al., 2021). Additionally, CHIP functions as a major degradation-promoting E3 ligase involved in quantitative control of Tat protein (Ali et al., 2019). In contrast, PJA2 promotes non-degradative polyubiquitination (K27, K29, K33-linked) and enhances transcriptional activity (Faust et al., 2017). Within the USP family, USP7 has been identified as a major stabilizing factor for Tat protein, protecting Tat protein from proteasomal degradation through removal of K48-linked ubiquitin chains (Ali et al., 2017). Meanwhile, USP21 inhibits HIV replication by downregulating Tat expression (Gao et al., 2021). The K19-specific Tat deubiquitination by USP45 identified in this study provides a new regulatory layer within this complex control network. Furthermore, our results demonstrate that this regulatory effect is strictly dependent on USP45’s catalytic activity. Given that multiple USP family members including USP7, USP21, and USP45 target Tat through distinct mechanisms, it is conceivable that these deubiquitinases cooperatively fine-tune Tat stability and function to dynamically regulate HIV-1 transcription and latency.

Interestingly, our screening revealed that while USP45 reduces Tat ubiquitination, other DUBs such as CDKN2A and ATXN3L paradoxically increased it. This may reflect complex regulatory mechanisms where these DUBs indirectly modulate E3 ligase activity or selectively target specific ubiquitin chain types, highlighting the diverse and context-dependent roles of DUBs in Tat regulation.

Analysis using latently infected model J-Lat 8.4 cells revealed that USP45 plays an important role in latency maintenance. Particularly, transcriptional profiling analysis using dPCR demonstrated that USP45 knockdown primarily promotes HIV-1 transcriptional initiation, which subsequently leads to enhanced downstream transcriptional processes including completion and post-transcriptional processing. Previous studies have shown that multiple stages of HIV-1 transcription are severely restricted in latently infected cells (Karn, 2011; Mbonye and Karn, 2011; Yukl et al., 2018). This is attributed to multiple factors including exclusion of key transcription factors from the nuclei of resting cells, hypoacetylated chromatin state of nucleosome-1, and recruitment of transcriptional repressors (Van Lint et al., 2013). Development of USP45-specific inhibitors could represent a new strategy for HIV-1 eradication therapy that complements the limitations of LRAs.

Considering that USP45 functions as an interferon-stimulated gene (ISG), sustained interferon responses may lead to high USP45 expression, which suppresses Tat activity, thereby limiting viral replication while promoting latency. However, type I interferon has been reported to contribute to both latency and reactivation in HIV-1 (Van der Sluis et al., 2020). This finding indicates the necessity of developing more precise therapeutic protocols that consider the balance between immune modulation and latent viral reactivation.

In summary, we have demonstrated that USP45 functions as a novel molecular switch for Tat in HIV-1 transcriptional initiation and viral latency. Such dynamic equilibrium between virus and host represents a promising therapeutic target for HIV-1 eradication therapy.

## Materials and methods

### Cell culture and transfection

HEK293T (ATCC, #CRL-1573) cells and Jurkat E6.1 (ECACC, #88042803) cells were maintained in Dulbecco’s Modified Eagle Medium (DMEM) and RPMI-1640 medium, respectively, supplemented with 10% fetal bovine serum (FBS) and 1% penicillin/streptomycin at 37 °C in a 5% CO_2_ atmosphere. J-Lat 8.4 cells (a latently HIV-1-infected Jurkat cell line) were obtained through the NIH AIDS Reagent Program (#9847) and cultured under the same conditions as Jurkat cells. Transient transfections were performed using Lipofectamine 3000 (Thermo Fisher Scientific) or Lipofectamine RNAiMAX (Thermo Fisher Scientific) according to the manufacturer’s instructions. USP45-specific siRNA and control siRNA were purchased from Qiagen (#SI02779273, SI02779280, SI03650318). For stable knockdown experiments, cells were transduced with lentiviral vectors expressing shRNA targeting USP45 (Santa Cruz Biotechnology, #sc-76859-V) or control shRNA (#sc-108080), followed by puromycin selection (1 μg/ml) for 7 days.

### Plasmids

The HIV-1 Tat expression vectors were constructed by subcloning the human-codon-optimized tat gene (synthesized by Eurofins genomics) into pcDNA6 with C-terminal HA tag or pNLF1-C with C-terminal NanoLuc luciferase, respectively. A library of 120 DUB-related gene expression vectors with HaloTag fusion were prepared by Kazusa DNA Research Institute and purchased from Promega. Detailed information for DUB constructs, including product IDs and sequences, is available at the Kazusa DNA Research Institute^1^ and summarized in Supplementary Table 1. Tat and USP45 mutants were generated using site-directed mutagenesis, and their sequences are provided in Supplementary Table 2. The ubiquitin expression vector was constructed by cloning the human-codon-optimized ubiquitin gene (synthesized by Eurofins genomics) into pBiT3.1-N (Promega). Luciferase expression constructs under the control of wild-type HIV-LTR (pLTR-luc) was described previously (Ryo et al., 2008).

### NanoBRET screening assay

NanoBRET assay was performed as described previously (Miyakawa et al., 2022; Miyakawa et al., 2022; Miyakawa et al., 2025). Briefly, HEK293T cells seeded in 96-well white plates were co-transfected with NanoLuc-fused Tat (1 ng) and individual HaloTag-fused DUB expression vectors (100 ng) from the 120 DUB library. After 24 h, cells were incubated with HT-618 Ligand (Promega) for 16 h, followed by addition of Nano-Glo Substrate (Promega). Bioluminescence resonance energy transfer (BRET) was measured using a GloMax Luminometer (Promega). BRET ratios were calculated as the ratio of acceptor emission (610 nm) to donor emission (460 nm). Assay validity was confirmed using positive and negative controls.

### Immunoprecipitation and immunoblotting analysis

HEK293T cells seeded in 6-well plates were co-transfected with vectors encoding HA-tagged Tat (100 ng) and HaloTag-fused DUB (100 or 200 ng) with or without HiBiT-tagged ubiquitin plasmids (100 ng). At 48 h post transfection, the cells were lysed in HBST buffer (10 mM HEPES pH 7.4, 150 mM NaCl, and 0.5% Triton-X-100) supplemented with protease inhibitors (Merck). For immunoprecipitation, cell lysates were incubated with anti-HA EZview Red Affinity Gel (Merck) overnight at 4 °C, and the bound proteins were washed three times with ice-cold HBST buffer and eluted by boiling in SDS sample buffer (Fujifilm-Wako). For immunoblotting analysis, proteins were separated by SDS-PAGE on 10–20% gradient gels (Fujifilm-Wako) and the proteins were blotted onto PVDF membranes (Merck). Membranes were blocked with Blocking One reagent (Nacarai) for 1 h at room temperature and probed with primary antibodies overnight at 4 °C, followed by HRP-conjugated secondary antibodies (Cytiva) for 30 min at room temperature. The detected proteins were visualized using a LuminoGraph imaging system (Atto). Primary antibodies used in this study included the following: USP45 (Atlas Antibodies, #HPA075668, 1:1000 dilution), HaloTag (Promega, #G9211, 1:1000 dilution), HA (MBL, #M180-3, 1:1000 dilution), HiBiT (Promega, #N7200, 1:1000 dilution), Ubiquitin-K48-specific (Millipore, #05–1307, 1:1000 dilution), Ubiquitin-K63-specific (Millipore, #05–1308, 1:1000 dilution), and α-Tubulin (MBL, #PM054-7, 1:5000 dilution).

### Luciferase reporter assays

HEK293T cells seeded in 12-well plates were co-transfected with HIV-1 LTR-Luc reporter plasmid (200 ng) and expression vector encoding Tat (200 ng) and USP45 (250 or 500 ng). After 48 h, luciferase activity was measured using the Bright-Glo Luciferase Assay System (Promega).

### HIV-1 production and replication assays

For viral production assays, HEK293T cells were transfected with HIV-1 molecular clone pNL4-3 (Adachi et al., 1986) along with USP45 expression vectors. Culture supernatants were collected 48 h post-transfection, and viral production was quantified by p24 ELISA (ZeptoMetrix). For replication assays, Jurkat T cells with stable USP45 knockdown were infected with HIV-1 at MOI 0.01 or 0.1. Viral replication was monitored by measuring p24 levels in culture supernatants over 7 days using ELISA.

### Measurement of HIV-1 transcripts

J-Lat 8.4 cells expressing control or USP45 shRNA were treated with TNFα (10 ng/ml, R&D Systems) or SAHA (1 μM, Sigma-Aldrich) for 24 h. DMSO was used as vehicle control. RNA was extracted from J-Lat 8.4 cells using TRIzol reagent (Thermo Fisher Scientific). Reverse transcription was performed using SuperScript III (Thermo Fisher Scientific) with random hexamer primers. PCR was performed using the QIAcuty digital PCR system (Qiagen) with stage-specific primer-probe sets targeting five HIV-1 transcripts: Read-through, TAR, LongLTR, PolyA, and Tat-Rev. Each viral transcripts were normalized with the amounts of RNaseP gene transcripts. Primer and probe sequences are described in Supplementary Table 3, based on previously published protocols (Kaiser et al., 2017; Yukl et al., 2018; Telwatte et al., 2019).

### Interferon treatment and qPCR analysis

HEK293T and Jurkat T cells were treated with 1 μg/ml of IFN-α2A, IFN-β, IFN-γ, or IFN-λ1 (Proteintech) for 24 h. Total RNA was extracted and reverse transcribed as described above. Quantitative PCR was performed using TB Green Master Mix (Takara) on a StepOnePlus Real-Time PCR System (Applied Biosystems). USP45 mRNA levels were normalized to ACTB expression using the ΔΔCt method. Primer sequences are described in Supplementary Table 3.

### Statistical analysis

Statistical significance was determined using unpaired Student’s t-test for two-group comparisons. *p*-values < 0.05 were considered statistically significant. Statistical analyses were performed using GraphPad Prism 10 software.

## Data Availability

The raw data supporting the conclusions of this article will be made available by the authors, without undue reservation.

## Footnotes

1
https://www.kazusa.or.jp/kop/dsearch-e/
