# Supplementary material for: USP45-mediated deubiquitination of HIV-1 Tat regulates viral transcription and latency
Source: Front Microbiol. 2026 Jan 26;17:1656512. doi: 10.3389/fmicb.2026.1656512 (PMC12883740; doi:10.3389/fmicb.2026.1656512)
Supplement: Supplementary file 1 [file Data_Sheet_1.pdf]

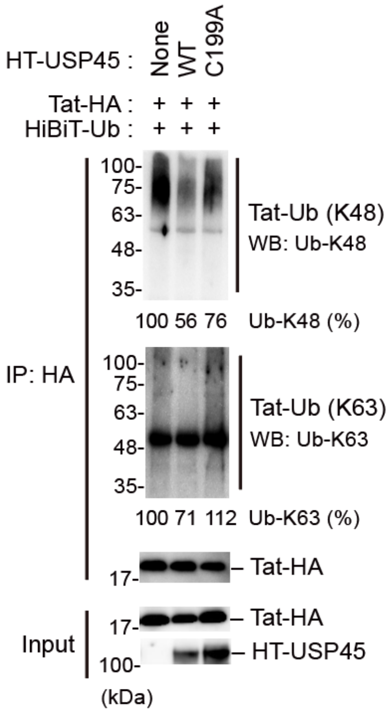

Supplementary Figure S1. USP45 targets both K48-linked and K63-linked ubiquitin chains on Tat protein.

HEK293T cells were co-transfected with HA-tagged Tat, HiBiT-tagged ubiquitin, and either wild-type USP45 or catalytically inactive USP45 C199A mutant. Tat ubiquitination was assessed by immunoprecipitation with anti-HA antibody followed by Western blotting using antibodies against K48-linked and K63-linked ubiquitin.

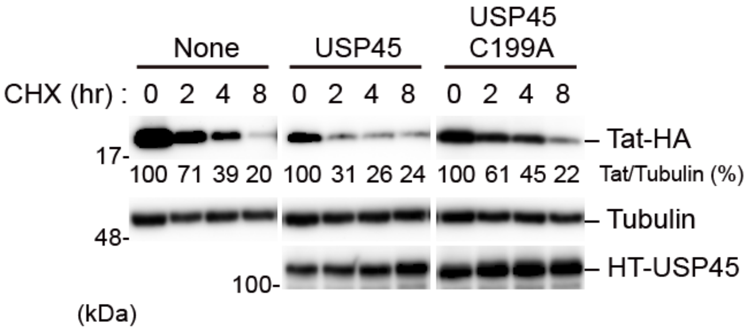

Supplementary Figure S2. USP45 accelerates Tat protein degradation in a catalytic activity-dependent manner.

Cycloheximide chase assay in HEK293T cells transfected with Tat-HA alone or with USP45 wild-type or C199A mutant. Cells were treated with cycloheximide (100 μg/ml), and samples were collected at indicated time points. Tat protein levels were analyzed by immunoblotting. Numbers indicate relative Tat expression normalized to 0 hours. Tubulin serves as loading control.

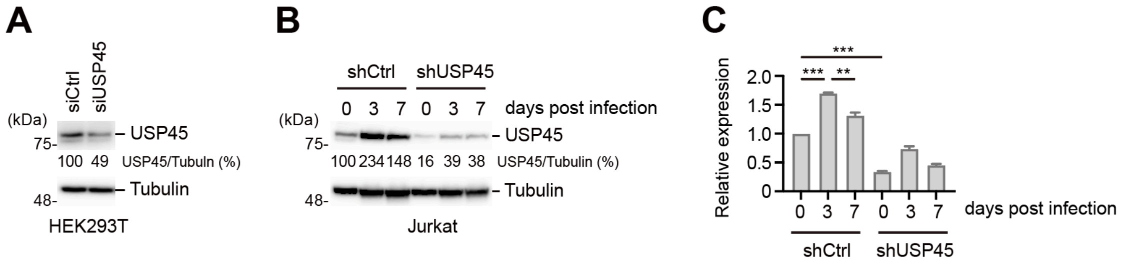

Supplementary Figure S3. USP45 expression during HIV-1 infection.

(A) USP45 knockdown efficiency in HEK293T cells.

(B, C) USP45 protein expression (B) and mRNA levels (C) during HIV-1 infection time course. Jurkat cells with stable shCtrl or shUSP45 were infected with HIV-1 (MOI 0.1). Data are mean ± SD (n=3). $^{**}P < 0.01$, $^{***}P < 0.001$.

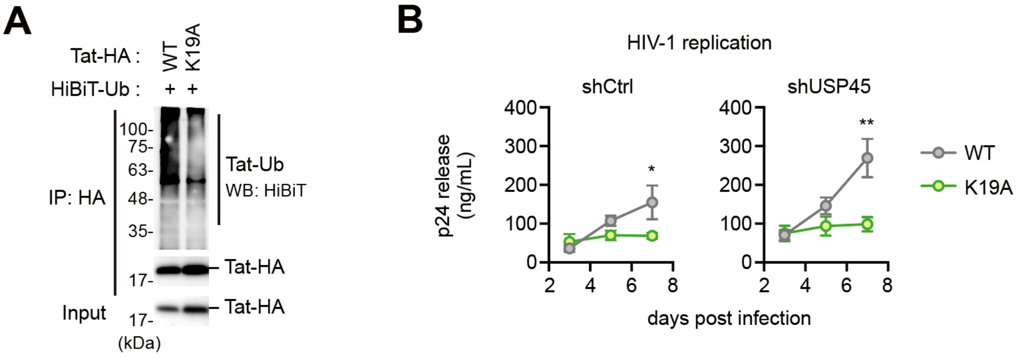

Supplementary Figure S4. Tat K19 is a major ubiquitination site and required for USP45-mediated HIV-1 restriction.

(A) HEK293T cells were transfected with wild-type Tat-HA or K19A mutant together with HiBiT-Ubiquitin. Cell lysates were immunoprecipitated with anti-HA antibody, and ubiquitinated Tat was detected by Western blot using anti-HiBiT antibody.

(B) Replication kinetics of wild-type and K19A mutant HIV-1 in USP45 knockdown cells. shCtrl or shUSP45 Jurkat cells were infected with WT or K19A mutant virus (MOI 0.1), and p24 release was measured at indicated timepoints. Data are mean ± SD (n=3). **P* < 0.05, ***P* < 0.01.

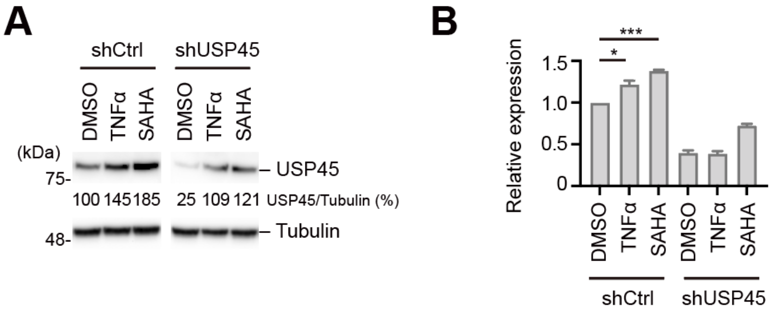

Supplementary Figure S5. Latency reversing agents induce USP45 expression.
(A) Protein and **(B)** mRNA levels of USP45 in shCtrl or shUSP45 J-Lat cells treated with DMSO, TNFα, or SAHA for 24 hours. Numbers in (A) indicate relative USP45/tubulin ratio (%). Data are mean ± SD (n=3). *P < 0.05, ***P < 0.001.
